# Retardation of the Calvin Cycle Contributes to the Reduced CO_2_ Assimilation Ability of Rice Stripe Virus-Infected *N. benthamiana* and Suppresses Viral Infection

**DOI:** 10.3389/fmicb.2019.00568

**Published:** 2019-03-20

**Authors:** Ji’an Bi, Yong Yang, Binghua Chen, Jinping Zhao, Zhuo Chen, Baoan Song, Jianping Chen, Fei Yan

**Affiliations:** ^1^Center for Research and Development of Fine Chemicals, Guizhou University, Guiyang, China; ^2^The State Key Laboratory Breeding Base for Sustainable Control of Pest and Disease, Key Laboratory of Biotechnology in Plant Protection of MOA of China and Zhejiang Province, Institute of Virology and Biotechnology, Zhejiang Academy of Agricultural Sciences, Hangzhou, China; ^3^Institute of Plant Virology, Ningbo University, Ningbo, China

**Keywords:** rice stripe virus, phosphoribulokinase, glucose, the Calvin cycle, viral infection

## Abstract

Rice stripe virus (RSV) is naturally transmitted by the small brown planthopper and infects plants of the family *Poaceae*. Under laboratory conditions, RSV can infect *Nicotiana benthamiana* by mechanical inoculation, providing a useful system to study RSV–plant interactions. Measurements of CO_2_ assimilation ability and PSII photochemical efficiency showed that these were both reduced in *N. benthamiana* plants infected by RSV. These plants also had decreased expression of the *N. benthamiana Phosphoribulokinases* (*NbPRKs*), the key gene in the Calvin cycle. When the *NbPRKs* were silenced using the TRV-Virus Induced Gene Silencing system, the plants had decreased CO_2_ assimilation ability, indicating that the downregulated expression of *NbPRKs* contributes to the reduced CO_2_ assimilation ability of RSV-infected plants. Additionally, *NbPRKs*-silenced plants were more resistant to RSV. Similarly, resistance was enhanced by silencing of either *N. benthamiana Rubisco* small subunit (*NbRbCS*) or *Phosphoglycerate kinase* (*NbPGK*), two other key genes in the Calvin cycle. Conversely, transgenic plants overexpressing *NbPRK1* were more susceptible to RSV infection. The results suggest that a normally functional Calvin cycle may be necessary for RSV infection of *N. benthamiana*.

## Introduction

Rice stripe virus (RSV), the type member of the genus *Tenuivirus*, causes chlorotic striping on the leaves of rice plants leading to significant yield losses in East Asia, including China, Japan, and Korea. The virus is naturally transmitted by the small brown planthopper (*Laodelphax striatellus* Fallén), and only infects plants of the family *Poaceae*. The RSV genome consists of four single-stranded RNAs and four of the seven ORFs identified are translated from an intermediate complementary sense RNA (Zhu et al., 1991, 1992; Hamamatsu et al., 1993; Takahashi et al., 1993; Qu et al., 1997; Wu et al., 2013). It is therefore difficult to construct infectious clones for reverse genetics experiments and studies have been limited to exploring the pathogenesis of RSV and its interaction with plants or the insect vectors (which are also hosts of the virus). However, using mechanical inoculation in the laboratory, RSV can infect *Nicotiana benthamiana* and this provides a useful system for studying the interactions between RSV and plants (Xiong et al., 2008; Zhang et al., 2012). RSV-infected *N. benthamiana* plants are stunted and their leaves become twisted with chlorotic mottling.

The process of photosynthesis in plants converts light energy to chemical energy, in the form of the energy molecules ATP and NADPH, that are used for the Calvin cycle to fix CO_2_. The Calvin cycle, also known as the Calvin–Benson cycle, is a series of biochemical redox reactions that take place in the stroma of chloroplasts in photosynthetic organisms. This set of reactions finally converts carbon dioxide and water into organic sugars. Phosphoribulokinase (PRK) is an essential enzyme in the Calvin cycle. It catalyzes phosphorylation of ribulose 5-phosphate (RuP) into ribulose 1,5-bisphosphate (RuBP), the initial substrate and CO_2_-acceptor molecule. PRK is unique to the Calvin cycle, and its activity often determines the carbon fixation rate.

Previously we found that nine chloroplast-related genes (ChRGs) were downregulated in RSV-infected *N. benthamiana* and showed that their silencing caused leaf chlorosis, indicating an association of ChRGs with RSV-induced chlorosis (Shi et al., 2016). We have now investigated the interaction between RSV and photosynthesis in more detail and show that RSV-infected *N. benthamiana* plants have reduced CO_2_ assimilation ability and PSII photochemical efficiency. Downregulation of *N. benthamiana PRK* (*NbPRKs*) was found to contribute to this reduction. Furthermore, silencing of *NbPRKs*, and either of *N. benthamiana Rubisco* small subunit (*NbRbCS*) or *Phosphoglycerate kinase* (*NbPGK*), two other key genes in the Calvin cycle, inhibited RSV infection, while overexpression of *NbPRK1* facilitated infection. The results suggest that a normally functional Calvin cycle may be necessary for RSV infection of *N. benthamiana*.

## Results

### CO_2_ Assimilation Ability and PSII Photochemical Efficiency Were Decreased in RSV-Infected Plants

**FIGURE 1:**
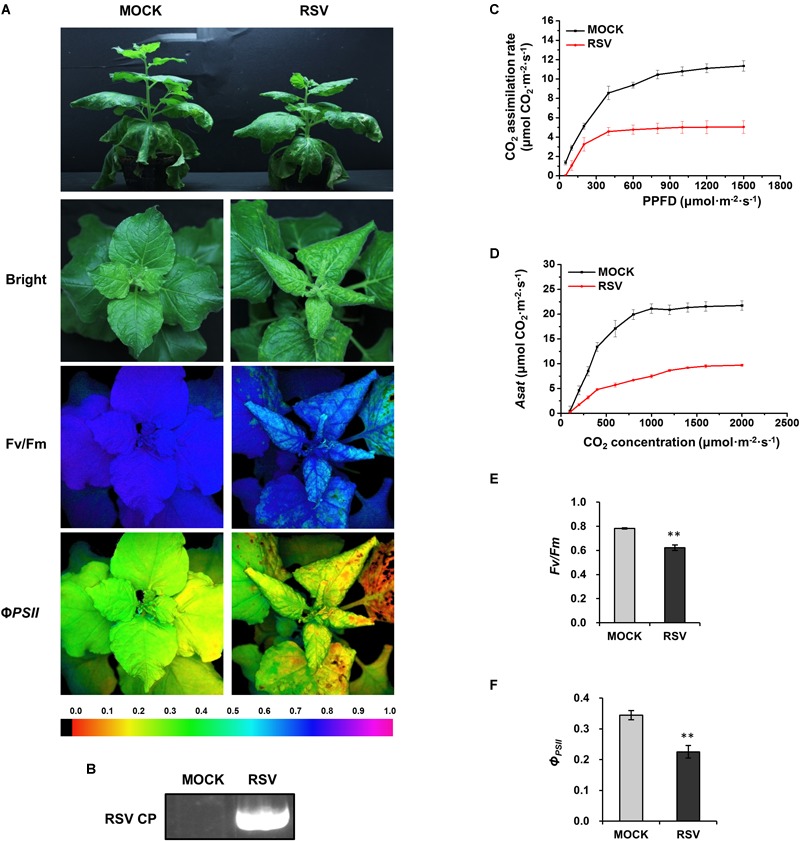
CO_2_ assimilation and PSII photochemical efficiency were decreased in RSV-infected *N. benthamiana.* **(A)** Mock and RSV-infected plants under bright light and the corresponding fluorescence images used to estimate PSII maximum photochemical quantum yield (*F_v_/F_m_*) and effective photochemical quantum yield (*Φ_PSII_*). The false color code depicted at the bottom of the image ranged from 0 (black) to 1.0 (purple). **(B)** Results of RT-PCR confirming the presence of the RSV CP gene in plants inoculated with RSV. **(C)** Light response curve showing that the CO_2_ assimilation rates at different photosynthetic photon flux densities (PPFD) were significantly decreased in RSV-infected leaves. **(D–F)** The light-saturated CO_2_ assimilation rate (*Asat*) (D) *F_v_/F_m_* (E) and *Φ_PSII_* (F) were all significantly reduced in RSV-infected plants compared to the mock-inoculated control plants. Results in **(C–F)** are from three independent replicates (three plants were examined for each replicate). Bars represent the standard errors of the means. A two-sample unequal variance directional *t*-test was used to test significance of the difference (^∗∗^*p* < 0.01).

*N. benthamiana* plants inoculated with RSV 20 days earlier were systemically infected; they were stunted and their leaves were twisted with chlorotic mosaic symptoms suggesting that photosynthesis had been affected (Figure 1A,B). Compared to mock-inoculated controls, plants infected by RSV had significantly lower rates of CO_2_ assimilation at different photosynthetic photon flux densities (PPFD; Figure 1C). There were also decreases in the light-saturated CO_2_ assimilation rate (*Asat*; Figure 1D), PSII maximum photochemical quantum yield (*F_v_/F_m_*; Figure 1E) and effective photochemical quantum yield (*Φ_PSII_*; Figure 1F). All these results indicate that RSV infection suppressed CO_2_ assimilation and PSII photochemical efficiency in *N. benthamiana*.

### Expression of *NbPRK* mRNAs Was Downregulated in RSV-Infected Plants

In a previous study, we used a GeneChip^®^Tomato Genome Array to identify changes in gene expression following infection of *N. benthamiana* by RSV (Shi et al., 2016). Among the differentially expressed genes (DEGs) with two-fold expression difference, we identified an EST highly similar to *Arabidopsis thaliana PRK* gene (At1g32060) that was downregulated in infected plants. Because PRK is an essential enzyme for photosynthesis in the Calvin cycle, we have now examined this further.

**FIGURE 2:**
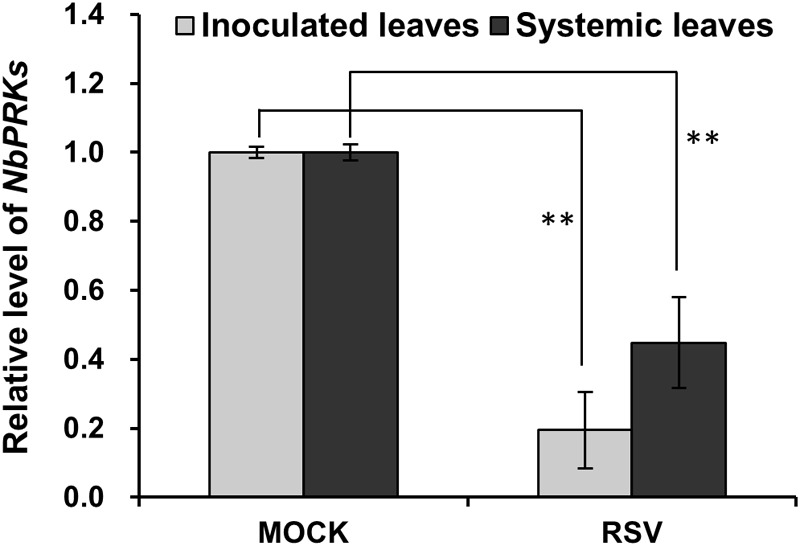
Expression of *NbPRK* mRNAs was downregulated in RSV-infected *N. benthamiana*. qRT-PCR demonstrating the downregulated expression of *NbPRKs* in RSV-inoculated leaves at 7 dpi and RSV-systemically infected leaves at 14 dpi. Results are from three independent replicates (six plants for each replicate, three technical replicates for each plant). Bars represent the standard errors of the means. A two-sample unequal variance directional *t*-test was used to test significance of the difference (^∗∗^*p* < 0.01).

The *PRK* genes of *N. benthamiana* were first identified from the *N. benthamiana* genome v1.0.1 predicted cDNA database^1^ using *A. thaliana PRK* gene as query, and these were then cloned. Two sequences with 73.9% and 74.4% nucleotide identity to *AtPRK*, and 82.8% and 81.5% amino acid identity to AtPRK, respectively, were identified and named as *NbPRK1* (ID: Niben101Scf10910g00006.1) and *NbPRK2* (ID: Niben101Scf12623g00008.1). The expression of these genes 7 days after inoculation with RSV was then tested by qRT-PCR using mock-inoculated leaves as a control. Because the two sequences were so similar (97.2% nt and 96.6% aa identity), it was not possible to design primers to distinguish them (Supplementary Table S1) and the results therefore reflect the joint expression of *NbPRK1* and *NbPRK2*. The expression of *NbPRKs* in RSV-inoculated leaves was only 10–30% of that in the control leaves (Figure 2). In the newly emerged systemically infected leaves collected at 14 dpi, the expression of these genes was 20–50% of control levels (Figure 2). Thus RSV infection downregulated the expression of *NbPRKs*.

### Silencing of *NbPRKs* Blocked CO_2_ Assimilation and Decreased PSII Photochemical Efficiency

**FIGURE 3:**
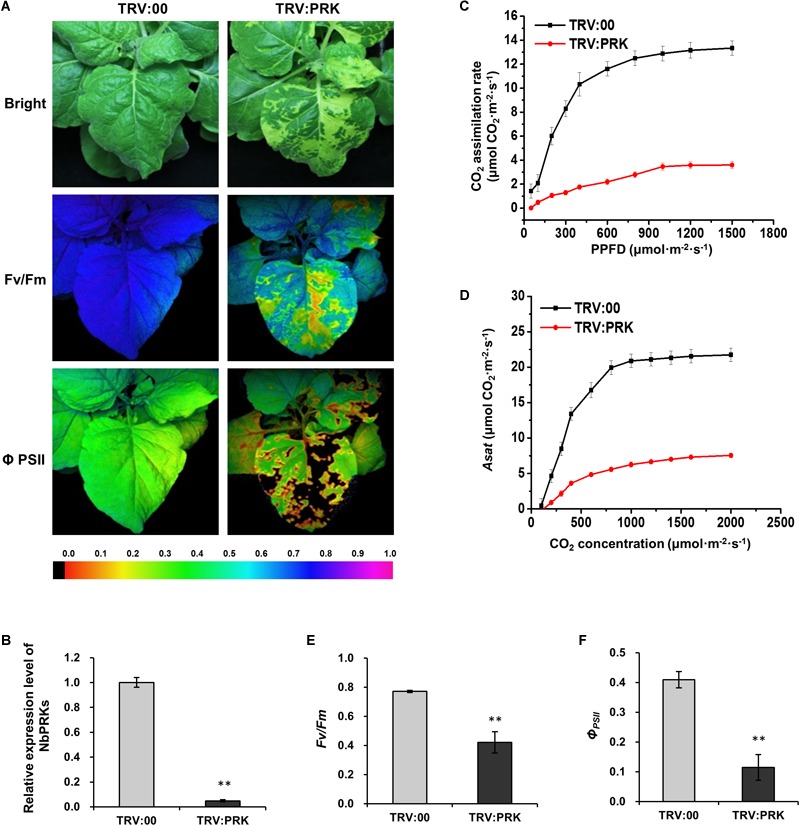
Silencing of *NbPRKs* decreased CO_2_ assimilation and PSII photochemical efficiency. **(A)** TRV:00-treated (control) and TRV:PRK-treated (*NbPRKs*-silenced) plants under bright light and the corresponding fluorescence images used to estimate PSII maximum photochemical quantum yield (*F_v_/F_m_*) and effective photochemical quantum yield (*Φ_PSII_*). The false color code depicted at the bottom of the image ranged from 0 (black) to 1.0 (purple). **(B)** Results from qRT-PCR showing that the expression of *NbPRKs* in TRV:PRK-treated plants was 10% of that in non-silenced controls. Results are from three independent replicates (six plants for each replicate, three technical replicates for each plant). **(C)** The CO_2_ assimilation rates at different photosynthetic photon flux density (PPFD) were remarkably reduced in *NbPRKs*-silenced plants. **(D)** The *Asat* was notably reduced in *NbPRKs*-silenced plants. **(E,F)** *F_v_/F_m_* **(E)** and *Φ_PSII_* **(F)** were significantly decreased by *NbPRKs* silencing. Results in **(C–F)** are from three independent replicates (three plants were examined for each replicate). Bars represent the standard errors of the means. A two-sample unequal variance directional *t*-test was used to test significance of the difference (^∗∗^*p* < 0.01).

To investigate the effect of downregulation of *NbPRKs* on plants, we used the tobacco rattle virus-induced gene silencing (VIGS) system to silence *NbPRKs*. At 10 dpi of VIGS treatment, the expression of *NbPRKs* was down to 10% of that in non-silenced plants infected with empty TRV vector (TRV:00) (Figure 3B). The silenced plants had obvious mottle chlorosis on their newly emerged leaves (Figure 3A). The CO_2_ assimilation rates at different PPFD were remarkably reduced in silenced plants and there was also a reduction in *Asat* (Figure 3C,D). To determine whether the photochemical efficiency was also affected by silencing of NbPRK, we measured the *F_v_/F_m_* and *Φ_PSII_* in silenced plants and found that both were significantly decreased compared to the controls (Figure 3E,F). Thus, silencing of NbPRKs suppressed the plant’s photochemical efficiency.

### Silencing of *NbPRKs* Inhibited RSV Infection

**FIGURE 4:**
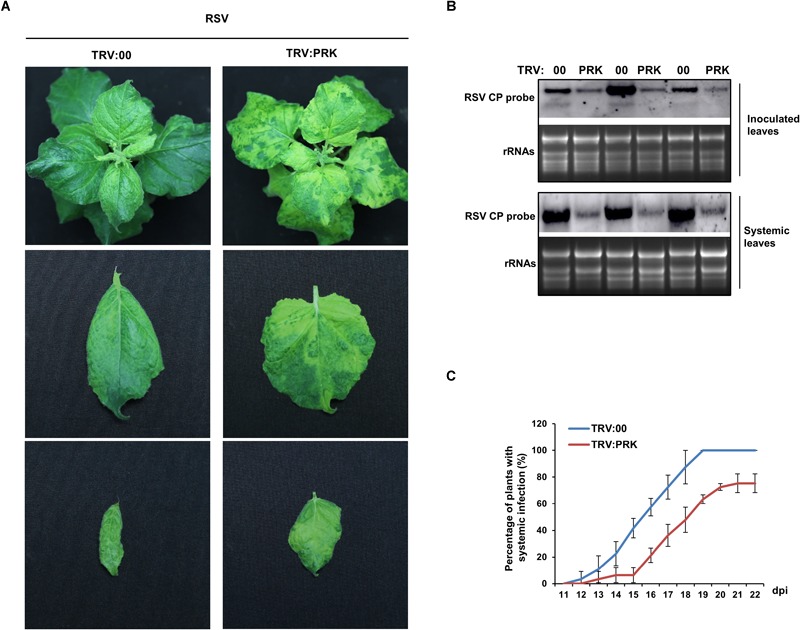
Silencing of *NbPRKs* inhibited RSV infection. **(A)** The leaf-twisting symptom on *NbPRKs*-silenced plants was much milder than that on the non-silenced plants inoculated with empty TRV vector (TRV:00). **(B)** Northern blotting showing that RSV RNAs had accumulated at lower levels in *NbPRKs*-silenced plants than in non-silenced plants in both the inoculated leaves at 7 dpi and the systemically infected leaves at 15 dpi. Results of three replicates are shown. **(C)** Percentage of plants systemically infected with RSV at different times after inoculation; values are consistently smaller on silenced plants than on non-silenced plants. Data from three replicates (at least 20 plants per replicate) were analyzed.

Next, to investigate the effect of the downregulation of *NbPRKs* on RSV infection, we inoculated RSV onto *NbPRKs*-silenced plants. At 15 dpi, typical symptoms appeared on both silenced and control plants. However, the leaf-twisting symptoms on silenced plants were much milder than those on the controls treated with empty TRV vector (Figure 4A). To exclude the possibility that the TRV vector might affect virus symptoms, we compared the symptoms of RSV on TRV:00-treated plants and on mock-treated plants, and found no obvious difference (Supplementary Figure S1). In the inoculated leaves (at 7 dpi), there were fewer RSV RNAs on the silenced than on control plants (Figure 4B). At 15 dpi, leaves of plants systemically infected by RSV were sampled from both treatments to detect the accumulation of RSV RNAs in them. Consistent with the results from the inoculated leaves, there were fewer RSV RNAs in systemically infected leaves of the silenced plants than in the controls (Figure 4B). The development of systemic infection on the newly emerged leaves from 11 dpi was also slower on the silenced plants (Figure 4C). These results show that silencing of *NbPRKs* inhibited RSV infection.

### Silencing of Other Genes in the Calvin Cycle (*RbCS* or *PGK*) Also Inhibited RSV Infection

**FIGURE 5:**
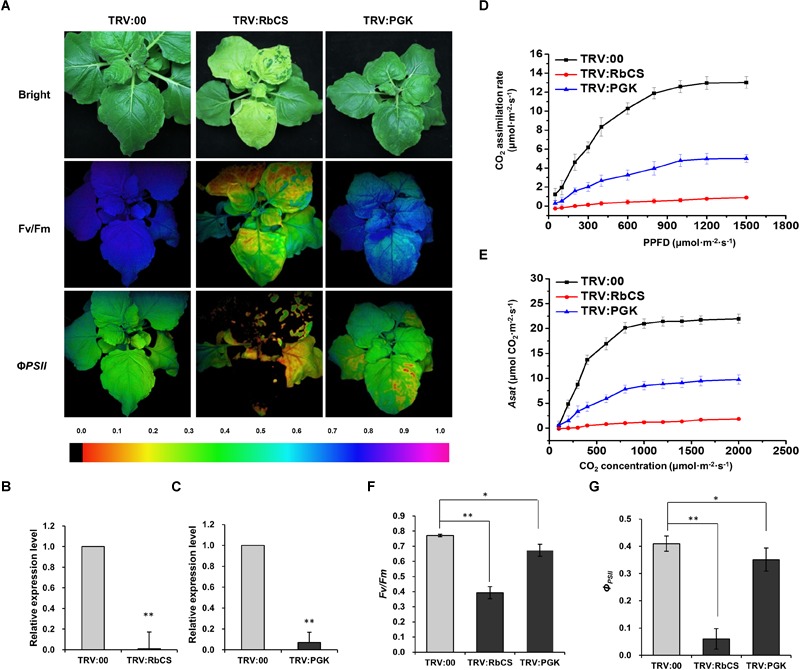
Silencing of *NbRbCS* or *NbPGK*, two key genes in the Calvin cycle, also decreased CO_2_ assimilation and PSII photochemical efficiency. **(A)** TRV:00-treated (control), TRV:RbCS and TRV:PGK-treated plants under bright light and the corresponding fluorescence images used to estimate *Fv/Fm* and *Φ_PSII_*. The false color code depicted at the bottom of the image ranged from 0 (black) to 1.0 (purple). **(B,C)** qRT-PCR showing the relative expression levels of *NbRbCS* **(B)** and *NbPGK* **(C)** in TRV:RbCS-treated and TRV:PGK-treated plants. Results are from three independent replicates (six plants for each replicate, three technical replicates for each plant). **(D–G)** Significant reductions in the CO_2_ assimilation rates at different PPFDs **(D)**, *Asat* **(E)**, *F_v_/F_m_* **(F)** and *Φ_PSII_* **(G)** in plants where *NbRbCS* or *NbPGK* had been silenced. Results are from three independent replicates (three plants were examined for each replicate). Bars represent the standard errors of the means. A two-sample unequal variance directional *t*-test was used to test significance of the difference (^∗^*p* < 0.05; ^∗∗^*p* < 0.01).

**FIGURE 6:**
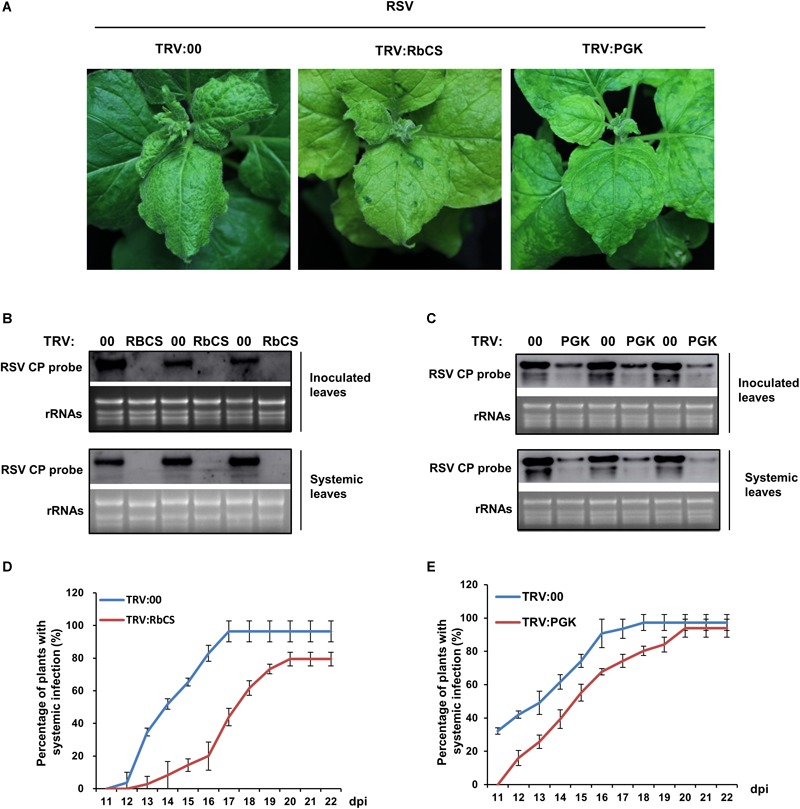
Silencing of *NbRbCS* or *NbPGK* inhibited RSV infection on *N. benthamiana*. **(A)** The viral symptoms (leaf twisting and chlorosis) were alleviated on both *NbRbCS* and *NbPGK*-silenced leaves compared to those on the control. **(B,C)** Northern blotting showing that RSV RNAs had accumulated less in *NbRbCS-* **(B)** and *NbPGK*-silenced **(C)** plants than in non-silenced controls in both the inoculated leaves at 7 dpi and the systemically infected leaves at 14 dpi. **(D,E)** Percentage of plants systemically infected with RSV; values are lower when *NbRbCS-* **(D)** or *NbPGK*- **(E)** were silenced compared to non-silenced plants. Data from three replicates (at least 20 plants per replicate) were analyzed. Bars represent the standard errors of the means.

Since PRK is a key enzyme in the Calvin cycle, we next investigated whether retarding the Calvin cycle in other ways would also affect RSV infection. We therefore silenced two other key genes in the Calvin cycle, *N. benthamiana RbCS* (*NbRbCS*) and *PGK* (*NbPGK*), and investigated the susceptibility of these plants to RSV. Silencing of *NbRbCS* significantly reduced CO_2_ assimilation and PSII photochemical efficiency, and also caused plant chlorosis (Figure 5), while silencing of *NbPGK* reduced CO_2_ assimilation and PSII photochemical efficiency slightly (Figure 5). On both sets of silenced plants at 14 dpi the viral symptoms of twisted-leaf and chlorosis were less than on the control (Figure 6A). Northern blotting showed that RSV RNAs had accumulated less in both the inoculated and RSV-systemically infected leaves of the silenced plants (Figure 6B,C). Additionally, the percentage of the plants systemically infected with RSV remained lower on the silenced plants (Figure 6D,E). These results suggest that the retardation of the Calvin cycle might explain the increased RSV resistance of *NbPRK*-silenced plants, and may also indicate that RSV infection requires the Calvin cycle to function normally.

### Overexpression of *NbPRK1* Facilitated RSV Infection

**FIGURE 7:**
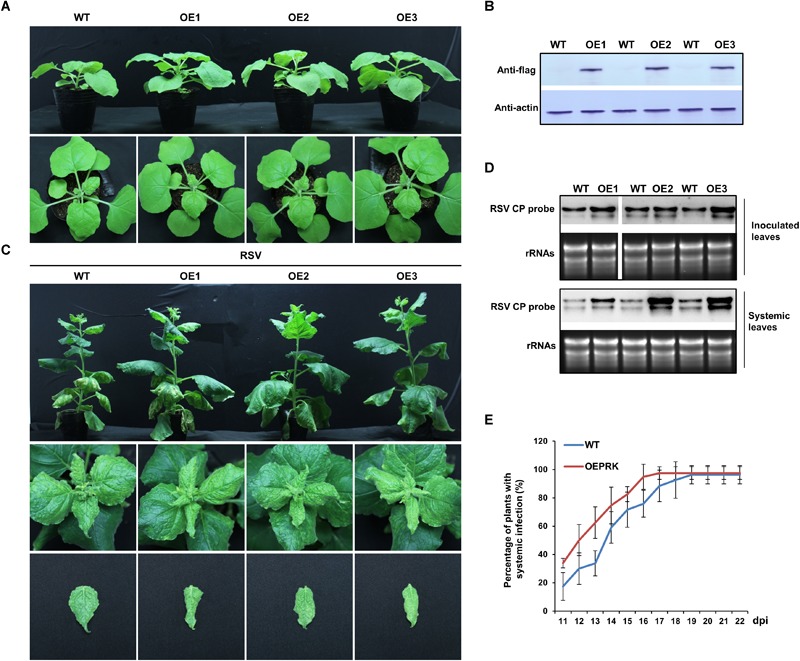
Overexpression of *NbPRK1* facilitated RSV infection of *N. benthamiana.* **(A)** Plants of three independent T2 transgenic lines (OE1, OE2, and OE3) showing their normal phenotype. **(B)** Flag-fused NbPRK1 protein was expressed more in the transgenic lines than in the wild type control. **(C)** The leaf-twisting symptoms on plants overexpressing *NbPRK1* were much more severe than those on wild type plants. Leaves have been pinned down to show the twisting symptoms. **(D)** RSV RNAs accumulated more in both the inoculated and the systemically infected leaves of transgenic plants. **(E)** The time course of systemic infection showing that transgenic plants continued to be less frequently infected than wild type plants. Data from three replicates (at least 20 plants per replicate) were analyzed.

To further understand the relationship between *NbPRKs* and RSV infection, we next overexpressed Flag-fused *NbPRK1* in *N. benthamiana* and investigated RSV infection on the transgenic plants. The T2 transgenic plants had a normal phenotype (Figure 7A) and the overexpression of Flag-fused NbPRK1 protein was confirmed in three independent T2 transgenic lines (OE1, OE2, and OE3) (Figure 7B). After inoculation with RSV, both transgenic and wild type plants showed typical symptoms at 15 dpi, but the leaf-twisting symptoms on plants overexpressing *NbPRK1* were much more severe than those on wild type plants (Figure 7C). The expression levels of *NbPRKs* mRNAs in RSV-infected transgenic OE lines were higher than those in mock wild type plants (Supplementary Figure S2). Northern blotting using samples of inoculated leaves at 7 dpi showed a much greater accumulation of RSV RNAs in the transgenic plants and there were similar results from testing systemically infected leaves at 15 dpi (Figure 7D). The percentage of plants systemically infected with RSV remained greater on transgenic plants than on the wild type controls (Figure 7E). We also analyzed the CO2 assimilation ability, and the PSII photochemical efficiency in RSV-infected transgenic OE lines, but found no obvious difference to RSV-infected wild type plants (Supplementary Figure S3). We think that the large accumulation of RSV in transgenic OE lines must aggravate the symptoms by other routes.

## Discussion

The experiments reported here describe the effect of RSV infection on CO_2_ assimilation and photochemical efficiency, and demonstrate that downregulation of *NbPRKs* is involved in that effect. Silencing of *NbPRKs* decreased CO_2_ assimilation rate and photochemical efficiency, and caused chlorosis on the newly emerged leaves. In our earlier microarray experiments the decrease in expression of *NbPRKs* was not statistically significant (*p*-value 0.11) (Shi et al., 2016), while the qRT-PCR assay reported here indicated a significant downregulation in both RSV-inoculated leaves and systemic leaves (Figure 2). This suggests that the microarray data may yet be hiding other genes that are differentially expressed following RSV infection and will be worth exploring.

A virus uses enzymes, raw materials, and energy from its host to complete its life cycle. The final product of the Calvin cycle is sugar, the most important source of energy. Many human viruses rewire host cell glucose and glutamine metabolism to meet the bioenergetic and biosynthetic demands of viral propagation (Ritter et al., 2010; Vastag et al., 2011). Moreover, it has been reported that diversion of glucose carbon to fatty acid synthesis is essential for the success of human cytomegalovirus (HCMV) infection (Munger et al., 2008). Inhibition of fatty acid synthesis prevents the formation of infectious virions (Munger et al., 2008). Our results show that silencing of *NbPRKs, NbRbCS* or *NbPGK* reduced CO_2_ assimilation and that the silenced plants were less conducive to RSV infection (Figure 4, 6). We suppose that the retarded Calvin cycle in the silenced plants would decrease the levels of available sugar and that this might be unfavorable for RSV infection. Indeed, we confirmed that there were lower levels of both glucose and sucrose in plants where *NbPRKs, RbCS* or *PGK* had been silenced (Supplementary Figure S4). The results indicate that a normally functioning Calvin cycle may be necessary for RSV infection on the experimental host *N. benthamiana*. On rice, the natural host, RSV also causes chloroplast abnormalities and we have confirmed that the expression of *PRK, RbCS* and *PGK* genes in RSV-infected rice was downregulated (Supplementary Figure S5). This indicates that the Calvin cycle might play a similar role in both the experimental and natural hosts. It is known that different human viruses require different kinds of energy source (Ritter et al., 2010; Vastag et al., 2011). We do not know whether a functioning Calvin cycle is necessary for the infection of other plant viruses and this would be worth investigating.

Our results suggest that the downregulation of *NbPRKs* was a consequence of viral infection, contributing to the decreased CO_2_ assimilation rate and photochemical efficiency in RSV-infected plants. Meanwhile, the downregulation of *NbPRKs* also suppressed RSV infection, which indicates that the downregulation may perhaps be recognized as a defense response. This defense role may be a consequence of the decreased quantity of the final product of the Calvin cycle (Figure 4, 6). It is also possible that the decreased energy supplies might slow down the overall normal metabolic rate and genetic expression level, with knock on consequences for RSV infection. However, the NbPRKs-silenced plants did not show the full viral phenotype (only some chlorosis), suggesting that these general processes were not greatly affected and therefore may not play a major role in suppression of RSV infection.

Chloroplasts are the sites of photosynthesis with its light and Calvin cycle reactions. Of all the cellular organelles, it is the most significantly affected in function and ultrastructure by virus infection (Li et al., 2016; Budziszewska and Obrepalska-Steplowska, 2018; Jin et al., 2018). Increasing evidence indicates that proteins essential for the functioning of chloroplasts play negative roles in viral pathogenesis (Li et al., 2016; Zhao et al., 2016b; Budziszewska and Obrepalska-Steplowska, 2018; Jin et al., 2018). On the other hand, several chloroplast proteins are actually necessary for viral infection. For example, the Photosystem II oxygen evolution complex protein of *Nicotiana benthamiana* (NbPsbO1) interacts with 6K2 of Tobacco vein banding mosaic virus (TVBMV). Knockdown of NbPsbO1 significantly decreases the accumulation levels of TVBMV, as this chloroplast protein is hijacked to regulate potyvirus replication (Geng et al., 2017). *Nicotiana benthamiana* chloroplast Hsp70 (NbcpHsp70) family protein NbcpHsp70-2 was identified in complexes of Bamboo mosaic virus (BaMV) replicase. Silencing of NbcpHsp70-2 resulted in a significant decrease of BaMV RNA in *N. benthamiana* protoplasts, indicating that NbcpHsp70-2 is involved in the efficient replication of BaMV RNA (Huang et al., 2017). Our results suggest a further possible mechanism by which chloroplast proteins function in virus–plant interactions by manipulating the energy supplies to regulate viral infection.

In plant–microbe interactions generally, carbohydrates produced by photosynthesis are essential to fuel the energy required for defense. Increasing evidence also shows that sugar signals contribute to immune responses and probably depend greatly on coordinated relationships with hormones and light status (Bolouri Moghaddam and Van Den Ende, 2012; Trouvelot et al., 2014). Our results here show that silencing of *NbPRKs* led to a decreased concentration of glucose in plants, while improving the plant resistance against RSV. This suggests that there may be a glucose balance that deserves further analysis since it is an energy source that would promote RSV infection while it also plays a role as a signal priming plant defense.

## Materials and Methods

### Plant and Virus Inoculation

*N. benthamiana* plants were grown in a growth room at 24–26°C with a 16 h/8 h light/dark photoperiod cycle. Four-week-old plants with eight to nine leaves were used for TRV infiltration (Zhao et al., 2016a). Leaves were mechanically inoculated with crude extracts from RSV-infected rice leaves (carrying the RSV-Zhejiang isolate) at 10 dpi. The RSV-infected rice leaves were excised and homogenized in phosphate buffer using a sterile pestle and mortar. After centrifugation at 5,000 rpm for 2 min, 0.1 mL aliquots of the supernatant were then dropped onto leaves to inoculate them. Samples were collected from the infected plants, frozen and stored at -70°C until use (Wu et al., 2013).

### Virus-Induced Gene Silencing

TRV vectors containing pTRV1 and pTRV2-LIC were kindly provided by Dr. Yule Liu, Tsinghua University, Beijing, China (Liu et al., 2002). The partial sequence of *NbPRK* was amplified by primers NbPRK-Vigs-f and NbPRK-Vigs-r (sequences are shown in Supplementary Table S1), and then it was inserted into pTRV2-LIC producing TRV:PRKs to silence the internal *NbPRKs*. In a similar way, partial sequences of *NbRbCS* and *NbPGK* were amplified (using primers NbRbCS-VIgs-f and NbRbCS-VIgs-r for NbRbCS; NbPGK-VIgs-f and NbPGK-VIgs-r for NbPGK; see Supplementary Table S1) and were inserted into pTRV2-LIC producing TRV:RbCS and TRV:PGK to silence NbRbCS and NbPGK, respectively. TRV: 00 as an empty vector was used for the control treatment.

### Transformation of *N. benthamiana*

For transformation of *NbPRK1* to *N. benthamiana* plants, Flag-fused *NbPRK1* was cloned into the pCV vector with the CaMV 35S promoter and NOS terminator (Jiang et al., 2018). After inoculation of *N. benthamiana* leaf pieces with agrobacterium strain C58C1, transformed tissue was selected by culturing calli and then transformed plants were regenerated. Transformed plants were confirmed by qRT-PCR and the presence of NbPRK1-Flag protein was tested by Western blot using an antibody to Flag.

### RNA Extraction, RT-PCR, and qPCR

Total RNA was extracted using Trizol reagent (Invitrogen, Carlsbad, CA, United States) according to the manufacturer’s protocol. First-strand cDNA was synthesized from 1 μg of total RNA using oligo dT Primer and a random primer mix. DNA fragments corresponding to *UBC, NbPRK, NbRbCS, NbPGK*, RSV-CP were PCR amplified using their respective primers (see Supplementary Table S1) (Zhao et al., 2013).

The expression of *NbPRK, NbRbCS* or *NbPGK* was determined by real-time PCR using the respective primers. The Applied Biosystems^TM^ QuantStudio^TM^ 6 Flex Real-Time PCR System was used for the reaction and the results were analyzed by the ΔΔC_T_ method (Jiang et al., 2014). UBC was used as the reference gene in these experiments after preliminary tests with two other reference genes showed that the UBC expression level was not itself affected by RSV infection (results not shown).

### Northern Blot

For Northern blot analyses, a DNA probe targeting RSV-CP was synthesized and labeled with digoxin, pre-hybridization, hybridization and signal detection were performed according to the protocol of the DIG High Prime DNA Labeling and Detection Starter Kit II (Roche) (Jiang et al., 2018). The image was obtained using the Amersham Imager 600 System (GE, United States).

### Western Blot

Plant protein was extracted using extraction buffer (100 mM Tris-HCl, pH 8.8, 6% SDS and 2% 2-mercapto-ethanol), placed on ice for 30 min, centrifuged at 13,000 × *g* for 15 min and supernatant boiled at 100°C for 10 min. Protein samples were separated on 12% SDS-PAGE gels, followed by semi-dry electrophoretic transfer to Nitrocellulose membrane (GE Amersham protran 0.45 NC, United States). Blots were blocked with TBS buffer containing 5% skimmed milk and probed with the appropriate primary antibodies of anti-actin (Abbkine, CN) or anti-flag (Abbkine, CN). They were probed with anti-rabbit or anti-mouse alkaline phosphatase-conjugated secondary antibodies (Sigma). The immunoprobed proteins were visualized using NBT/BCIP buffer (Sigma).

### Measurements of Photosynthetic and Chlorophyll Fluorescence Parameters

CO_2_ assimilation rates at different PPFD and CO_2_ concentrations as well as *Asat* were measured by an LI-6400 Portable Photosynthesis System (LI-COR Inc., Lincoln, NE, United States). The youngest mature leaf was selected and tested at 9–11 a.m. The relative humidity was adjusted to approximately 60% by changing the parameters inside the measurement cuvette (Wang et al., 2004).

Images of chlorophyll fluorescence were obtained by an Imaging-PAM (IMAG-MAXI; Heinz Walz GmbH, Germany). The plants were kept in the darkroom for at least 30 min and measured quickly. There were three replicates for each treatment (Kong et al., 2014). ImagingWIN software (HeinzWalz GmbH, Germany) was used to monitor the value of *F_v_/F_m_* and *Φ_PSII_* at the continuous intermittent pulse intensity.

### Measurement of the Soluble Glucose in Chloroplasts

The plant chloroplasts were isolated using a chloroplast isolation kit (Sigma, St. Louis, MO, United States) according to the manufacturer’s protocol. After incubation in extraction buffer (0.3 mol/L HCl) overnight and centrifugation at 8,000 × *g* for 10 min at 4°C, the supernatant was transferred to a new tube and adjusted to pH 5 to 9. The samples were then filtered with syringe-type filters with pore size of 0.22 μm. To determine the glucose content, a HPLC method was established by using a 250 mm × 4.6 mm, Kro-masilNH2 column, ultrapure water as mobile phase with a flow rate of 0.4 mL⋅min^-1^, detector RID-10 and 80°C internal temperature.

## Data Availability

All datasets generated for this study are included in the manuscript and/or the Supplementary Files.

## Author Contributions

JC and FY conceived and designed the experiments. JB, YY, and BC performed the experiments. JB, YY, JZ, ZC, BS, JC, and FY analyzed the experimental data. JB, YY, JC, and FY wrote the manuscript.

## Conflict of Interest Statement

The authors declare that the research was conducted in the absence of any commercial or financial relationships that could be construed as a potential conflict of interest.

## Footnotes

1 https://Solgenomics.net/tools/blast

## Supplementary Material

FIGURE S1 Symptoms of RSV on TRV:00-treated plants and on mock treated plants.

FIGURE S2 Relative expression level of NbPRKs in RSV-infected OE lines (^∗∗^*p* < 0.01).

FIGURE S3 CO_2_ assimilation ability and the PSII photochemical efficiency in RSV-infected transgenic OE lines were shown without obvious difference to those in RSV-infected wild type plants. **(A)** Shows plants under bright light and the corresponding fluorescence images used to estimate PSII maximum photochemical quantum yield (*F_v_/F_m_*) and effective photochemical quantum yield (*Φ_PSII_*). The false color code depicted at the bottom of the image ranged from 0 (black) to 1.0 (purple). **(B,C)** Shows *F_v_/F_m_* **(B)** and *Φ_PSII_* **(C)** of plants. **(D,E)** Shows the CO_2_ assimilation rates at different photosynthetic photon flux density (PPFD) **(D)** and the *Asat* **(E)** of plants. OE lines were collected together for analysis in **(D,E)**. Results are from three independent replicates (three plants were examined for each replicate). Bars represent the standard errors of the means.

FIGURE S4 Glucose concentration in chloroplasts **(A)** and sucrose concentration in plants **(B)** decreased significantly in leaves where *NbPRKs, NbRbCS* or *NbPGK* had been silenced. Bars represent the standard errors of the means. A two-sample unequal variance directional *t*-test was used to test significance of the difference (^∗∗^*p* < 0.01).

FIGURE S5 **(A–C)** The expression of *PRK, RbCS* and *PGK* genes in RSV-infected rice was downregulated. Bars represent the standard errors of the means. A two-sample unequal variance directional *t*-test was used to test significance of the difference (^∗∗^*p* < 0.01).

TABLE S1 Primers used in this study.
