# Supplementary figures and images for: Retardation of the Calvin Cycle Contributes to the Reduced CO2 Assimilation Ability of Rice Stripe Virus-Infected N. benthamiana and Suppresses Viral Infection
Source: Front Microbiol. 2019 Mar 20;10:568. doi: 10.3389/fmicb.2019.00568 (PMC6435541; doi:10.3389/fmicb.2019.00568)

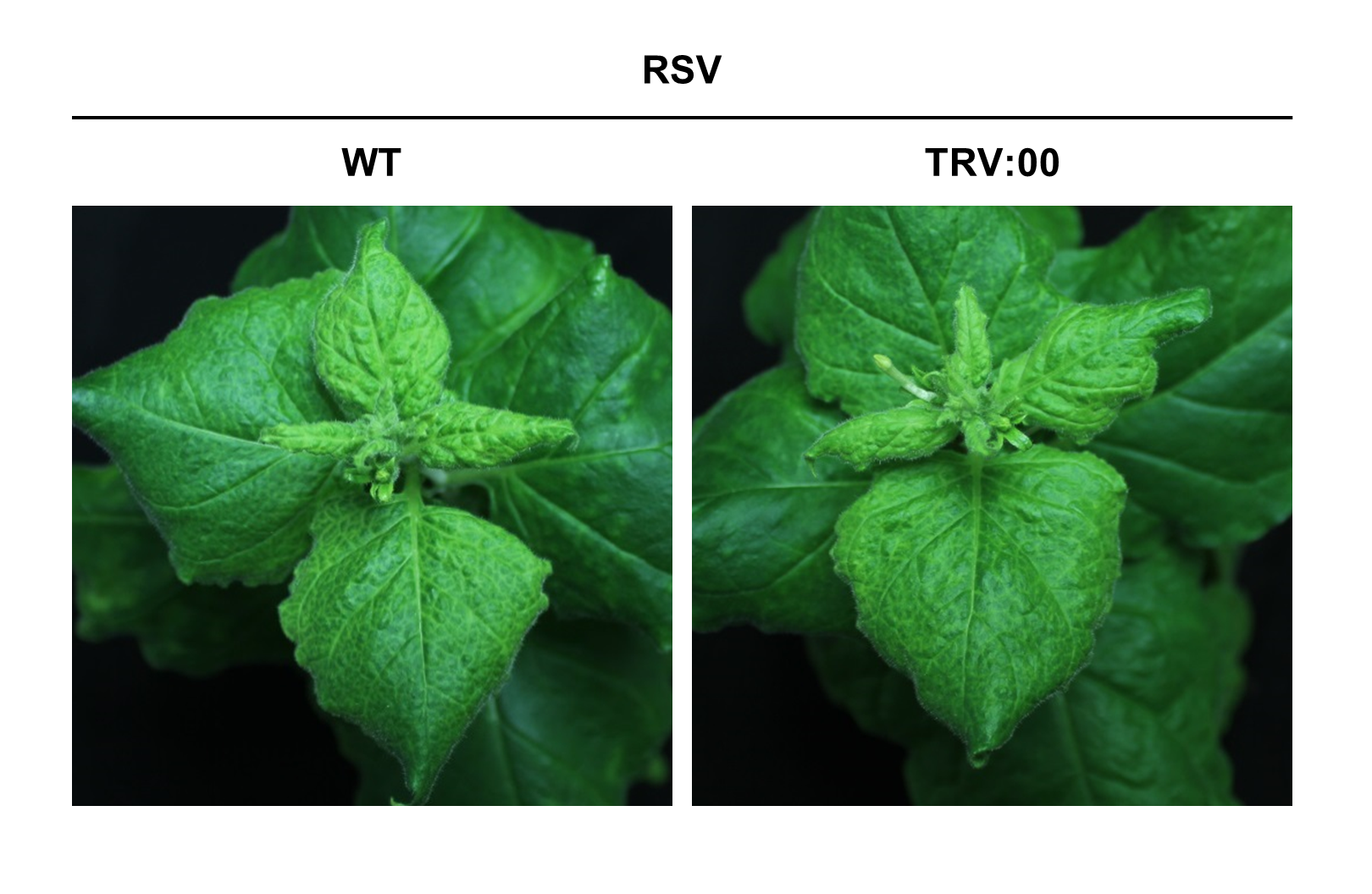

Supplement: FIGURE S1 — Symptoms of RSV on TRV:00-treated plants and on mock treated plants. [file Image_1.TIF]

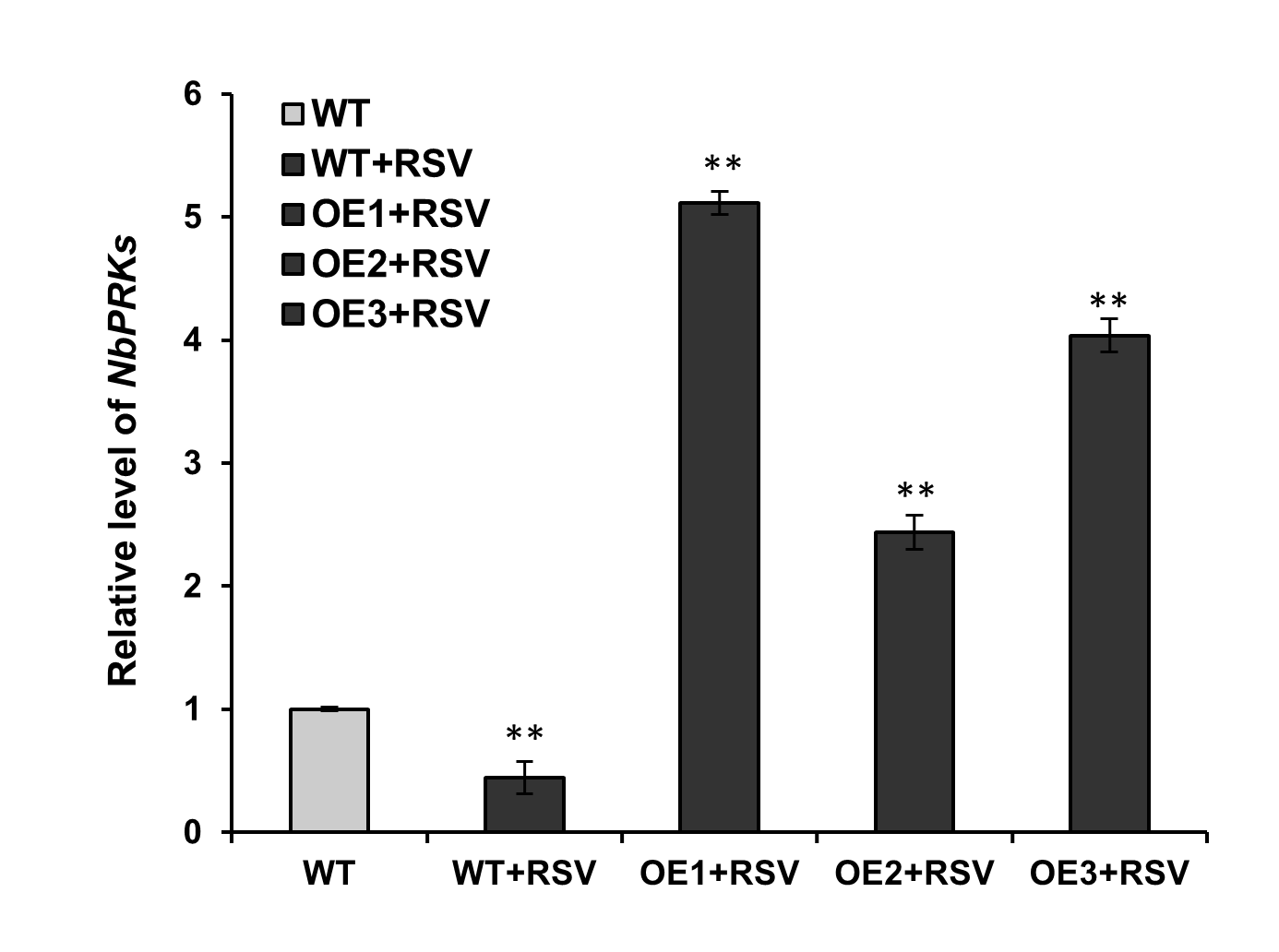

Supplement: FIGURE S2 — Relative expression level of NbPRKs in RSV-infected OE lines (∗∗p < 0.01). [file Image_2.TIF]

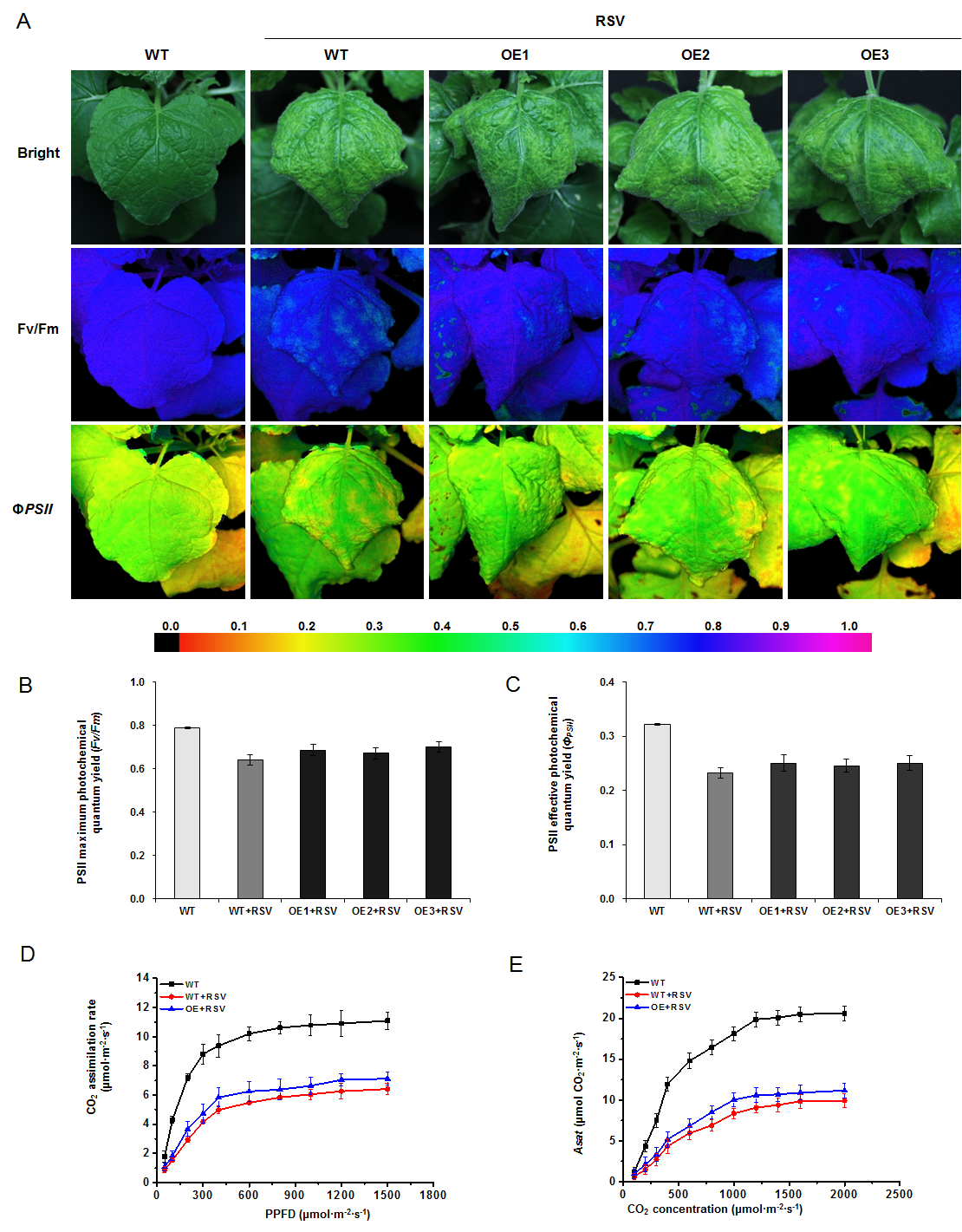

Supplement: FIGURE S3 — CO2 assimilation ability and the PSII photochemical efficiency in RSV-infected transgenic OE lines were shown without obvious difference to those in RSV-infected wild type plants. (A) Shows plants under bright light and the corresponding fluorescence images used to estimate PSII maximum photochemical quantum yield (Fv/Fm) and effective photochemical quantum yield (ΦPSII). The false color code depicted at the bottom of the image ranged from 0 (black) to 1.0 (purple). (B,C) Shows Fv/Fm (B) and ΦPSII (C) of plants. (D,E) Shows the CO2 assimilation rates at different photosynthetic photon flux density (PPFD) (D) and the Asat (E) of plants. OE lines were collected together for analysis in (D,E). Results are from three independent replicates (three plants were examined for each replicate). Bars represent the standard errors of the means. [file Image_3.TIF]

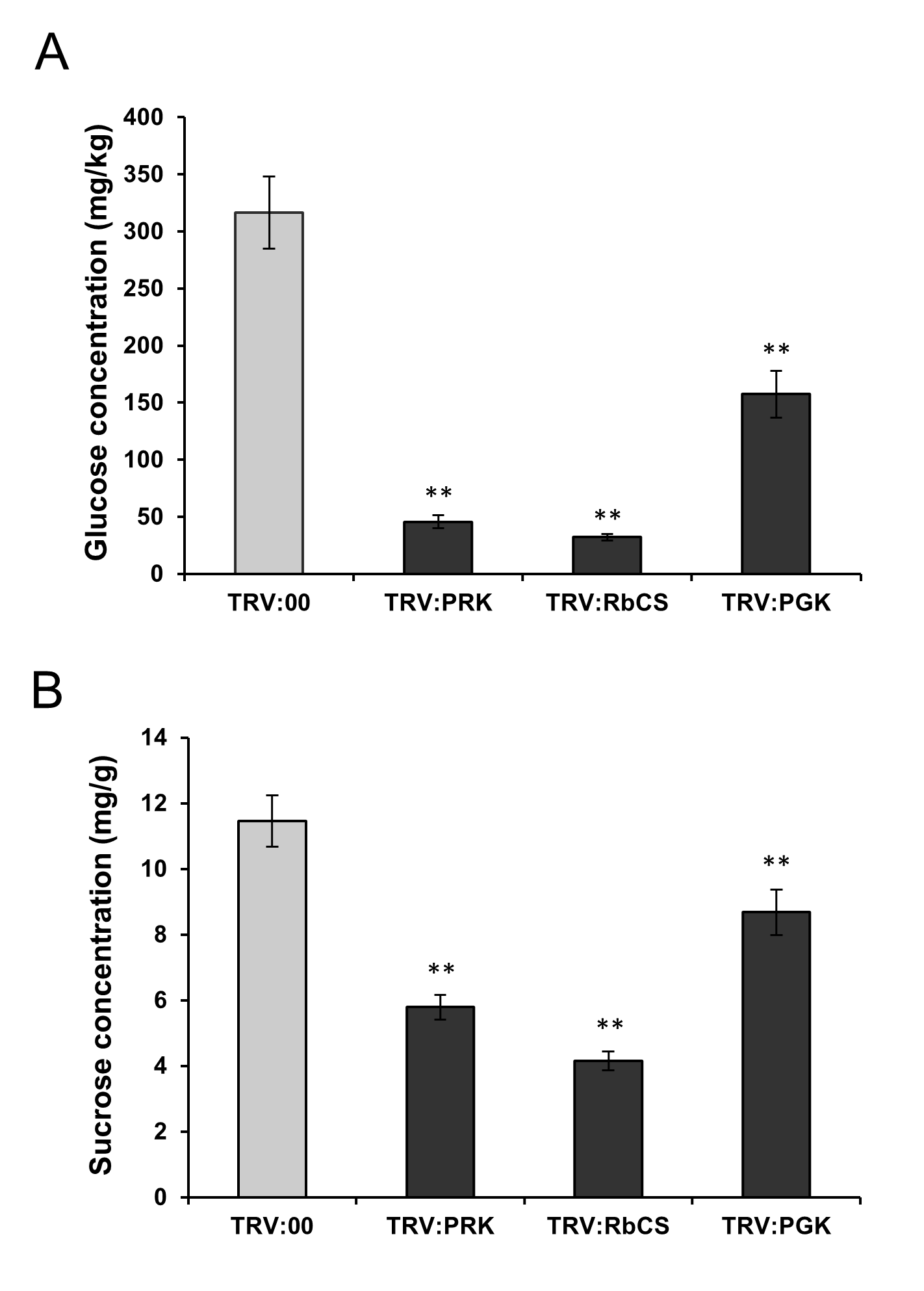

Supplement: FIGURE S4 — Glucose concentration in chloroplasts (A) and sucrose concentration in plants (B) decreased significantly in leaves where NbPRKs, NbRbCS or NbPGK had been silenced. Bars represent the standard errors of the means. A two-sample unequal variance directional t-test was used to test significance of the difference (∗∗p < 0.01). [file Image_4.TIF]

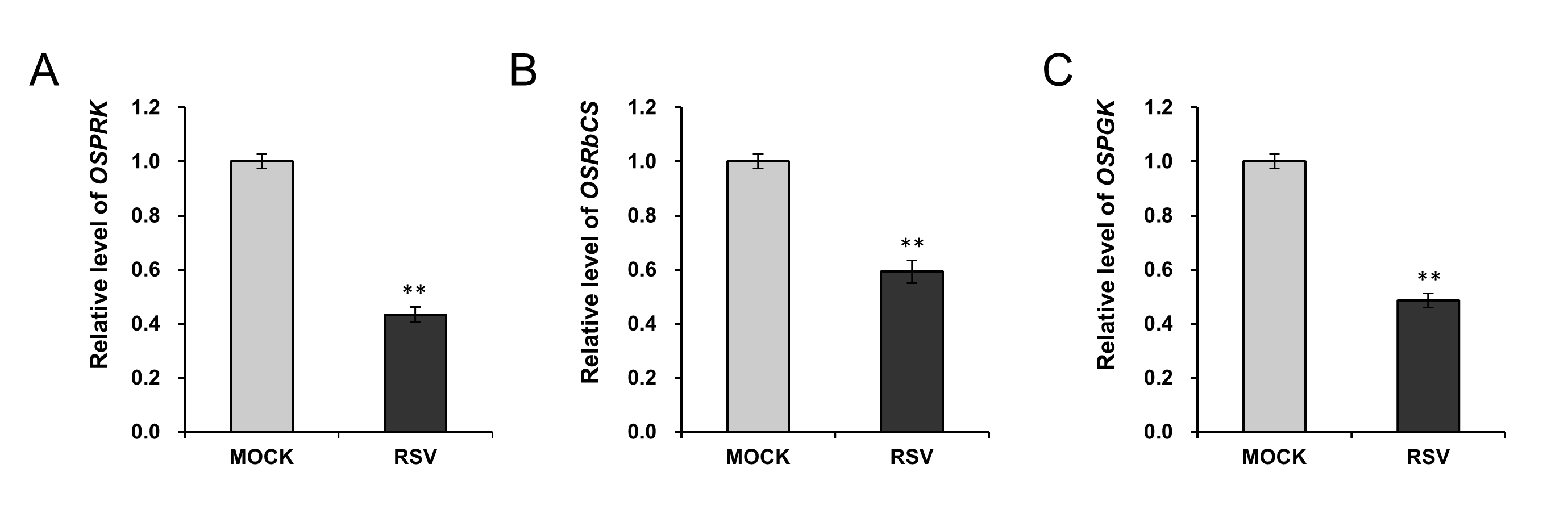

Supplement: FIGURE S5 — (A–C) The expression of PRK, RbCS and PGK genes in RSV-infected rice was downregulated. Bars represent the standard errors of the means. A two-sample unequal variance directional t-test was used to test significance of the difference (∗∗p < 0.01). [file Image_5.TIF]
