# Supplementary material for: Retardation of the Calvin Cycle Contributes to the Reduced CO2 Assimilation Ability of Rice Stripe Virus-Infected N. benthamiana and Suppresses Viral Infection
Source: Front Microbiol. 2019 Mar 20;10:568. doi: 10.3389/fmicb.2019.00568 (PMC6435541; doi:10.3389/fmicb.2019.00568)
Supplement: TABLE S1 — Primers used in this study. [file Table_1.docx]

**Table S1** Primers used in this study

| Name of the Primer | Sequence of the Primer (5’→3’) |
| --- | --- |
| NbPRK-Vigs-f  NbPRK-Vigs-r  NbPRK-qPCR-f  NbPRK-qPCR-r  NbRbCS-VIgs-f  NbRbCS-VIgs-r  NbRbCS-qPCR-f  NbRbCS-qPCR-r  NbPGK-VIgs-f  NbPGK-VIgs-r  NbPGK-qPCR-f  NbPGK-qPCR-r  NbUBC-f  NbUBC-r  RSVCP-Probe-f  RSVCP-Probe-r  NbPRKOE-f  NbPRKOE-r  NbAPR-f  NbAPR-r  NbEF1a-f  NbEF1a-r  OSPRK-qPCR-f  OSPRK-qPCR-r  OSRbCS-qPCR-f  OSRbCS-qPCR-r  OSPGK-qPCR-f  OSPGK-qPCR-r  OSActin-f  OSActin-r | CGACGACAAGACCGTCACCGACTACTGTGATTTGTCTTGATG  GAGGAGAAGAGCCGTCG GCCAGGGTAAGAACAAGTCAA  CTCCTGGTCATCACATGCAAG  GCCAGCGCATCGAAGATTGTG  CGACGACAAGACCGTCACC AATGCTGCTCAAGCCAACATG  GAGGAGAAGAGCCGTCG ATCCAATGATTCTAACCCAGGCT  TGGAGGCGATAAAACTGATG  GATGGGTTCCTTGCTTGG  CGACGACAAGACCGTCACC TCATCTGCTCGCTTCCTCG  GAGGAGAAGAGCCGTCG TCCTCCTTGTAGAATCTCACG  GGCAGTTTCAAATCCAAAGAG  CAACCAAGGAGGAACCAAC  TCTTGGAGATGGATGGGC  TCTGCTGGCAACGATTTG  TTTCGGTCCTGATGATACTCCC  CACAGAGCAAAGACTGGATTGA  CGACGACAAGACCGTCACC ATGGCAGTGAGTACAGTGTACA  GAGGAGAAGAGCCGTCG TTAGGCCTTTGCAGCTTGAACTG  CATCAGTGTCGTTGCAGGTATT  GCAACTTCTTGGGTTTCCTCAT  AGCTTTACCTCCCAAGTCATC  AGAACGCCTGTCAATCTTGG  GAGAGGTGACACAGCAAATG  CAGGTCTCTTCCAAACTATCG  GTGCCTTGCCTCGAGTTTAG  AAGCCGATGATGCGAACAAA  CCTGATGGTTGGATGGGTCT  GTAACACCCTTGCCGCTAAG  GGTATCCATGAGACTACATACAACT  TACTCAGCCTTGGCAATCCACAT |
